# Pharmacist burnout: from coping to system accountability in the medication-use process

**DOI:** 10.3389/fpubh.2025.1749332

**Published:** 2026-01-23

**Authors:** Esteban Zavaleta-Monestel, Lars-Åke Söderlund, Josep María Guiu-Segura, Jeaustin Mora-Jiménez, Sebastián Arguedas-Chacón

**Affiliations:** 1 Health Research Department, Clínica Bíblica, San José, Costa Rica; 2 Hospital Pharmacy Section, The International Pharmaceutical Federation, Hague, Netherlands; 3 The FIP Technology Advisory Group, The International Pharmaceutical Federation, Hague, Netherlands; 4 Faculty of Pharmacy and Food Sciences, University of Barcelona, Barcelona, Spain; 5 Department of Pharmacy, Clínica Bíblica, San José, Costa Rica

**Keywords:** burnout, health-system resilience, patient safety, pharmacist, workforce wellbeing

## Introduction

Burnout has escalated from an individual occupational challenge to a workforce-level crisis that threatens the medication-use process and the stability of health systems. Across community, hospital, and primary-care settings, pooled prevalence estimates indicate that nearly one in two pharmacists experience significant emotional exhaustion (1). Evidence from diverse practice environments consistently shows that this burden arises primarily from structural and organizational pressures rather than individual vulnerability, reframing burnout as a systems-level outcome in contemporary pharmacy practice (2). Pharmacists consequently report some of the highest burnout rates among healthcare professionals, reflecting the growing complexity, intensity, and accountability embedded in their roles (3).

Modern pharmacy practice requires balancing clinical care with expanding administrative responsibilities and continuous interaction with digital systems. Pharmacists operate in interruption-prone environments while managing high-risk therapies, regulatory documentation, and performance metrics, often simultaneously. Pandemic-era workforce data further link staffing shortages, intensified workload, and limited recovery time to heightened burnout risk across settings (13). Empirical studies consistently identify excessive workload, time pressure, and constrained professional autonomy as key predictors of emotional exhaustion, depersonalization, and reduced intent to remain in the profession (4).

These conditions carry direct implications for patient safety. Fatigue, cognitive overload, and frequent interruptions diminish the sustained attention required for accurate dispensing, verification, and patient counseling, thereby increasing the likelihood of preventable medication errors (5). As pharmacists' cognitive and moral capacity becomes strained, the reliability of the medication-use process itself is compromised. Recognizing burnout as an organizational and system-design issue is therefore essential for protecting both workforce wellbeing and the integrity of patient care.

This Opinion Article argues that pharmacist burnout should be understood not merely as a concern for personal wellbeing but as a signal of misaligned system design, with direct implications for patient safety and health-system resilience. Unlike recent narrative and systematic reviews that primarily describe prevalence, correlations, or individual coping strategies, this article emphasizes organizational accountability and the design of the medication-use process as central determinants of workforce sustainability.

## Conceptual foundations of pharmacist burnout

Burnout in pharmacy is increasingly understood as the consequence of structural imbalance rather than a failure of individual resilience. Emotional exhaustion emerges when organizational systems create persistent mismatches between workload demands and available resources, rendering burnout a predictable outcome of how the medication-use process is designed and governed. Under these conditions, sustained cognitive strain and moral tension become embedded in daily practice. Protecting pharmacists' mental, emotional, and ethical capacities is therefore essential for maintaining safety in medication-use systems. Evidence demonstrates that human-centered workflow redesign can reduce waiting times, operational pressure, and medication near misses, underscoring the modifiability of these risks through system-level interventions (25).

Several established theoretical frameworks help clarify the mechanisms by which organizational design contributes to burnout. The Job Demands-Resources model posits that professional wellbeing depends on the balance between job demands, such as time pressure, workload intensity, interruptions, and emotional labor, and job resources, including autonomy, supervisory support, staffing adequacy, and recognition (6). When demands chronically exceed available resources, emotional exhaustion, disengagement, and diminished performance become increasingly likely outcomes rather than isolated events.

The Maslach Burnout Inventory (MBI) provides the dominant conceptual framework for measuring burnout across healthcare professions, defining it as three interrelated dimensions: emotional exhaustion, depersonalization, and reduced personal accomplishment (7). In pharmacy practice, this model captures the progressive erosion of professional meaning that occurs when pharmacists are unable to exercise clinical judgment, provide patient-centered care, or meet ethical obligations due to systemic constraints.

Sociotechnical Systems Theory further situates burnout within the broader design of work environments, emphasizing that workforce wellbeing is inseparable from the interaction between human actors, organizational structures, and technological systems (8). Within the medication-use process, fragmented digital platforms, poorly aligned automation, and interruption-prone workflows interact with staffing models and governance practices to amplify cognitive load and stress. From this perspective, burnout reflects system behavior rather than individual limitation.

**Figure 1:**
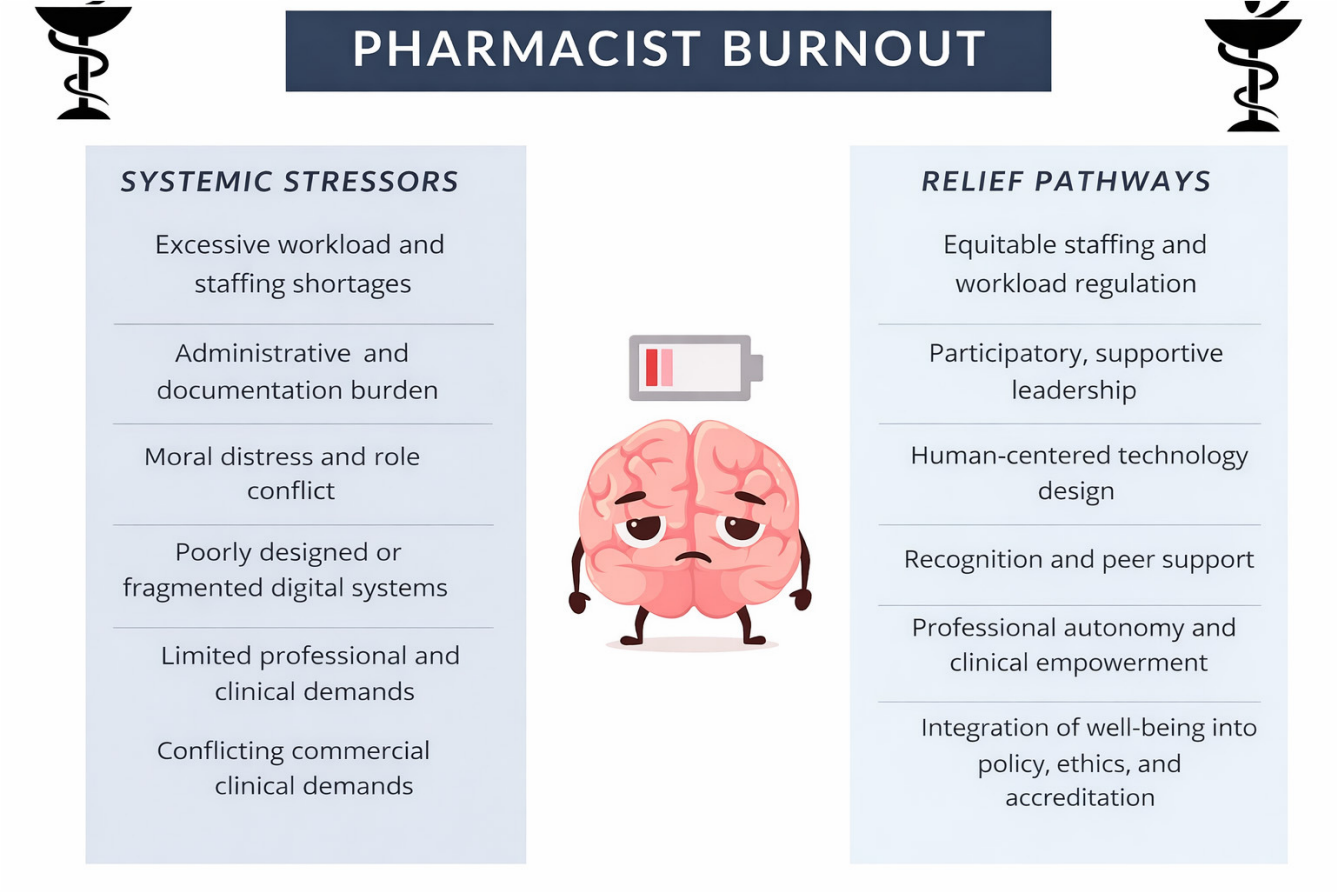
Systemic stressors, central role, and relief pathways in pharmacist burnout.

Collectively, these frameworks converge on the conclusion that pharmacist burnout is a structural and systemic phenomenon rooted in organizational design choices. They provide a conceptual foundation for understanding how workload intensity, technology, leadership, and performance metrics shape wellbeing across practice settings worldwide, as illustrated in Figure 1, and they establish the rationale for reframing burnout prevention as a matter of system accountability rather than individual endurance (9).

## Burnout across pharmacy practice settings

Pharmacist burnout has become a widespread occupational phenomenon spanning countries, sectors, and practice models. Although the specific drivers of exhaustion vary across environments, a shared imbalance between professional ideals and institutional design consistently undermines the meaning and sustainability of pharmaceutical care. This tension persists even as demand for safe, clinically engaged pharmacy services continues to rise globally.

In community practice, burnout frequently reflects structural compression driven by extended operating hours, limited staffing, and constant pressure to meet dispensing targets. National surveys consistently report high levels of emotional exhaustion among retail pharmacists, primarily attributed to workload intensity, insufficient recovery time, and commercial expectations that prioritize speed over clinical value (10). The resulting conflict between the professional obligation to protect patient safety and managerial demands for efficiency generates moral dissonance, a well-established predictor of burnout (11). Evidence from Greece illustrates this dynamic: nearly 60% of community pharmacists meet criteria for moderate-to-severe burnout, and autonomy and professional recognition are more strongly associated with job satisfaction than income. Comparable patterns are reported in Eastern Europe and the Gulf region, where studies from Bulgaria and Saudi Arabia link depersonalization and emotional exhaustion to understaffing, multitasking, and relentless prescription volume (12). Across diverse regulatory and cultural contexts, these findings converge on a consistent conclusion: commercial throughput erodes the moral foundation of community pharmacy practice (13).

Burnout within health-system pharmacies emerges through distinct mechanisms but carries comparable implications. Pharmacists responsible for medication safety operate within increasingly complex environments shaped by expanding compliance, documentation, and auditing requirements, all of which can weaken perceived professional control and autonomy (14). Digitalization, while intended to enhance safety, has introduced new sources of strain. Electronic health records, computerized provider order entry, and automated dispensing systems frequently generate excessive alerts, duplicative documentation, and fragmented workflows. These poorly integrated digital infrastructures contribute to attentional overload and cognitive fragmentation, thereby amplifying the risk of burnout (15).

The COVID-19 pandemic intensified pre-existing vulnerabilities within hospital settings. Pharmacists reported levels of emotional exhaustion comparable to those observed among nurses and physicians, often describing experiences of moral injury related to medication shortages, crisis-driven clinical decisions, and sustained exposure to patient deterioration (27). Post-pandemic assessments indicate persistent fatigue, emotional distancing, and reduced engagement, reflecting the cumulative psychological burden of prolonged crisis conditions.

In primary care and ambulatory settings, burnout often arises from the rapid expansion of clinical responsibilities without commensurate institutional support. Pharmacists increasingly contribute to chronic disease management, preventive services, and population health initiatives, yet frequently encounter role ambiguity, diffuse accountability, and inconsistent funding models (16). Administrative demands, including extensive documentation and performance reporting, have expanded alongside these clinical roles, diminishing professional autonomy and counteracting the intended benefits of team-based care integration.

Across all practice settings, a consistent pattern emerges. Burnout escalates when workload increases faster than organizational capacity. Interruption-prone workflows, competing priorities, and communication breakdowns have been empirically linked to both psychological strain and medication-safety events. Seminal observational studies demonstrate that each unscheduled interruption during dispensing increases the probability of a medication error. Subsequent research in Canadian hospital settings confirms that reducing workflow disruptions improves both dispensing accuracy and staff wellbeing (17, 18).

Despite contextual differences, the underlying design flaw remains consistent. Pharmacy systems are predominantly structured for throughput rather than sustainability. Whether processing high-volume retail prescriptions, managing high-risk therapies in hospitals, or reconciling complex medication regimens in primary care, speed remains the dominant performance metric. Core professional activities such as patient counseling, clinical reasoning, interprofessional collaboration, and mentoring are rarely captured in operational dashboards. This systematic undervaluation of cognitive labor reinforces environments that accelerate exhaustion and disengagement (17).

Intervention studies demonstrate that these pressures are not inevitable. Structural redesign strategies, including reallocating non-clinical tasks to support personnel, optimizing workflow sequencing, and institutionalizing protected clinical time, have been associated with reductions in near-miss events and improvements in job satisfaction (19). These findings reinforce a central principle: systems that generate burnout can also be redesigned to support professional resilience.

International literature consistently characterizes pharmacist burnout as a systemic imbalance between professional responsibility and institutional control. Across community, hospital, and ambulatory settings, environments that prioritize volume over value diminish workforce wellbeing and compromise vigilance (18). Recognizing emotional exhaustion as an organizational outcome rather than an individual failing is essential for aligning workloads, resources, and performance expectations with the ethical and safety foundations of the medication-use process.

## Systemic drivers and organizational reform

Burnout among pharmacists is increasingly recognized as a predictable consequence of structural imbalance rather than as a manifestation of personal fragility. Across practice settings, rising prescription volume, escalating productivity demands, and chronic understaffing reduce cognitive work to mechanical task execution (13). Post-pandemic workforce data identify unsustainable workload and inadequate recovery time as major predictors of emotional exhaustion. When institutional priorities consistently favor speed over clinical value, pharmacists experience moral and cognitive dissonance that erodes engagement and weakens the ethical foundations of practice (2).

Research across community and hospital environments reinforces these findings. Pharmacists frequently describe unrealistic performance expectations, limited autonomy, and persistent backlogs as central drivers of emotional exhaustion (13). These pressures manifest daily through prescription queues, staffing gaps, and unrelenting service demands. Under such conditions, pharmacists shift from reflective clinical decision-making to reactive task management, with insufficient time for counseling or complex therapeutic evaluation. Emotional detachment often emerges as a short-term coping response to sustained overload, but this disengagement diminishes vigilance, teamwork, and communication, increasing the risk of errors.

The economic consequences of these staffing failures further underscore their systemic nature. Multi-country analyses estimate that costs associated with turnover, absenteeism, recruitment, and productivity losses amount to billions of dollars annually (20). In North American hospital and retail settings, burnout is associated with increased sick leave and stronger intentions to leave the profession, undermining continuity of care and institutional memory (21). Pharmacy practice depends heavily on tacit knowledge and coordinated team performance. When turnover accelerates, this expertise is lost, directly affecting patient safety. Financial tools such as the ASHP Burnout Calculator illustrate that investment in workforce wellbeing functions as a cost-containment strategy by reducing turnover-related losses and strengthening operational efficiency, rather than serving as an optional wellness initiative (22).

## Technology and digital burden

Technology represents an additional and often underappreciated source of structural pressure. Although electronic health records, automated dispensing systems, and clinical decision-support tools were designed to enhance safety, poorly integrated digital environments frequently generate new forms of strain. Pharmacists working with fragmented or disjointed systems consistently report heightened stress and a diminished sense of control (23). Excessive alerts, repetitive data entry, and slow or unreliable software contribute to technological fatigue and compound cognitive overload.

Evidence from pharmacy informatics highlights that these burdens arise not from technology itself, but from inadequate human-centered design and insufficient alignment with real-world workflows (4). When digital tools fail to reflect the cognitive demands and sequencing of pharmacy work, automation can intensify burnout rather than alleviate it. Without deliberate integration, technology risks shifting rather than reducing workload.

## Leadership and governance practices

Leadership practices play a critical role in determining whether organizational conditions exacerbate or mitigate burnout. Studies involving pharmacy teams demonstrate that supportive leadership, characterized by transparency, recognition, and participatory decision-making, is consistently associated with lower levels of burnout (24). In contrast, authoritarian or narrowly metric-driven management approaches amplify alienation, stress, and emotional fatigue.

Leaders who acknowledge workload realities, involve staff in process redesign, and foster psychological safety help create environments in which professional wellbeing and performance reinforce one another. Training pharmacy managers in empathetic communication, staffing assessment, and workload planning has been associated with measurable improvements in retention and job satisfaction, confirming leadership as a structural determinant of safety rather than a supplementary interpersonal skill (24).

## Workflow redesign and protected clinical time

Evidence supporting structural redesign interventions is robust. Controlled evaluations of workflow optimization demonstrate improvements in waiting times, dispensing accuracy, and job satisfaction when administrative tasks are delegated and verification processes are streamlined (25). Reducing task fragmentation restores opportunities for sustained clinical attention, a resource as essential to medication safety as any technological safeguard.

Models that incorporate protected clinical time yield similar benefits by ensuring pharmacists can engage in medication review, patient counseling, and interprofessional collaboration without constant interruption. These interventions directly address the cognitive conditions necessary for safe practice and reinforce the principle that system design shapes professional behavior.

## Measurement, accountability, and performance metrics

International experiences demonstrate that meaningful reform is achievable when accountability structures are aligned with professional values. In Australia, national initiatives emphasizing balanced workloads and clinical contributions over transactional output have strengthened pharmacist satisfaction and performance (26). In Canada, primary-care frameworks that distribute medication-management responsibilities across interprofessional teams reduce duplication of effort and enhance collaborative satisfaction (27). These models share a common foundation: sustainable staffing and shared accountability support both workforce wellbeing and patient outcomes.

Emerging technologies such as artificial intelligence and predictive analytics may eventually relieve pharmacists of repetitive forecasting or screening tasks. However, evidence from high-volume community settings indicates that, without human-centered workflows, such technologies can exacerbate inefficiencies and introduce additional cognitive strain (28).

Underlying all successful initiatives is a fundamental shift in organizational ethos from endurance to accountability. Institutions that systematically monitor burnout and incorporate wellbeing indicators into performance dashboards report improvements in safety, engagement, and operational performance (29). Treating workforce wellbeing metrics with the same priority as error rates or clinical outcomes enables early intervention and supports proactive system governance. Transparency around workload, interruptions, and staffing levels transforms organizational learning from a reactive process into a continuous one.

Reimagining productivity is central to this transition. Measuring success primarily through prescriptions dispensed per hour undervalues cognitive labor and clinical judgment (30). Meaningful productivity reflects accuracy, quality of counseling, continuity of care, and patient outcomes. When institutional metrics prioritize these dimensions, efficiency and ethical practice become mutually reinforcing, restoring alignment between professional purpose and organizational expectations.

The trajectory of reform points toward pharmacy systems designed with human sustainability at their core. Adequate staffing, integrated technology, and compassionate leadership are not optional enhancements but structural prerequisites for safe medication use. Organizations that invest in these domains consistently demonstrate lower burnout, fewer errors, and higher patient satisfaction. As health systems adapt to post-pandemic realities and evolving patient needs, aligning institutional policies with evidence on pharmacist wellbeing remains essential for sustaining a resilient and ethically grounded medication-use process.
